# A multiomics dataset for the study of RNA modifications in human macrophage differentiation and polarisation

**DOI:** 10.1038/s41597-024-03076-8

**Published:** 2024-02-28

**Authors:** Natalia Pinello, Renhua Song, Quintin Lee, Emilie Calonne, Mark Larance, François Fuks, Justin J. -L. Wong

**Affiliations:** 1 Epigenetics and RNA Biology Program Centenary Institute, The University of Sydney, Camperdown, 2050 Australia; 2 Faculty of Medicine and Health, The University of Sydney, Camperdown, 2050 Australia; 3 Laboratory of Cancer Epigenetics, Faculty of Medicine, ULB Cancer Research Center (U-CRC), Jules Bordet Institute, Université Libre de Bruxelles (ULB), Brussels, Belgium; 4 Charles Perkins Centre, School of Medical Sciences, The University of Sydney, Camperdown, 2050 New South Wales Australia

**Keywords:** Transcriptomics, Myelopoiesis, RNA modification, Proteomics

## Abstract

RNA modifications have emerged as central regulators of gene expression programs. Amongst RNA modifications are N6-methyladenosine (m^6^A) and RNA 5-hydroxymethylcytosine (5hmC). While m^6^A is established as a versatile regulator of RNA metabolism, the functions of RNA 5hmC are unclear. Despite some evidence linking RNA modifications to immunity, their implications in gene expression control in macrophage development and functions remain unclear. Here we present a multi-omics dataset capturing different layers of the gene expression programs driving macrophage differentiation and polarisation. We obtained mRNA-Seq, m^6^A-IP-Seq, 5hmC-IP-Seq, Polyribo-Seq and LC-MS/MS data from monocytes and resting-, pro- and anti-inflammatory-like macrophages. We present technical validation showing high quality and correlation between samples for all datasets, and evidence of biological consistency of modelled macrophages at the transcriptomic, epitranscriptomic, translational and proteomic levels. This multi-omics dataset provides a resource for the study of RNA m^6^A and 5hmC in the context of macrophage biology and spans the gene expression process from transcripts to proteins.

## Background & Summary

RNA modifications are reversible chemical changes introduced to RNA molecules during or following their synthesis, which potentially regulate RNA metabolism and fate. More than 170 RNA modifications have been described so far and together they constitute the ‘epitranscriptome’^1,2^. The epitranscriptome introduces an additional layer for gene expression regulation in fundamental biological processes.

N-6 methyladenosine (m^6^A), is the most abundant internal RNA modification to mRNA in mammals^3,4^. It is deposited by a methyltransferase or ‘writer’ complex comprising a catalytic subunit, METTL3^5–8^ and several auxiliary proteins^9–12^. m^6^A methylation is reversed by m^6^A ‘erasers’, FTO^13^ and ALKBH5^14^. RNA binding proteins called m^6^A ‘readers’ recognise m^6^A-methylated RNAs to activate downstream processes based on environmental cues^15–19^. As an essential player in physiological processes, m^6^A regulates cell development^20^, maintains cellular identity^21^ and modulates key processes like the immune response^22–24^. Aberrant m^6^A methylation is therefore implicated in diverse human diseases^25–27^.

RNA 5-hydroxymethylcytosine (5hmC) is generated through the oxidation of 5-methylcytosine (5mC) by the Ten-eleven translocation proteins:TET1, TET2 and TET3^28–30^. RNA 5hmC regulates infection-induced myelopoiesis^31^, the fate of endogenous retroviral transcripts^32^ and pluripotency-associated transcripts stability^30^. Compared to m^6^A^3,33–35^, only a few studies have investigated transcriptome-wide RNA 5hmC functions^29,30^.

Macrophages provide the first immunological barrier to invading pathogens. Present in all tissues, these sentinel cells can integrate multiple environmental signals and execute critical functions including detecting pathogens or danger signals, phagocytosis, antigen presentation, secreting pro-inflammatory mediators and producing factors to resolve inflammation^36–38^. Besides executing critical inflammatory and immunomodulatory functions, macrophages are involved in various biological processes such as tissue repair and remodeling, iron homeostasis, modulating reactive oxygen species levels and other immunometabolic functions^39–41^.Thanks to their plastic nature, macrophages can transition from ‘basal’ or ‘resting’ states characteristic of homeostasis to ‘polarised’ pro- or anti-inflammatory states. Macrophage polarisation is a highly dynamic process and refers to different states adopted in response to particular stimuli, at a given time and within a specific context or microenvironment^42,43^. Broadly, macrophages can be differentially polarised to pro- or anti-inflammatory-like states, commonly referred to as M1- or M2-like macrophages, respectively. While classical M1 macrophages typically present enhanced cytotoxicity, secrete pro-inflammatory cytokines (like IL-1β, IL-6 and TNF), and execute antimicrobial functions; alternatively-activated M2 macrophages present an anti-inflammatory phenotype with roles in fibrosis, wound healing and the resolution of inflammation^38,44^. Due to the enormous heterogeneity characteristic of macrophages, the dichotomous M1/M2 paradigm fails to represent the system’s complexity. However, while acknowledging the limitations, the M1/M2 classification provides a valuable framework for studying molecular mechanisms driving macrophage functions within the context of selected immune stimuli^45,46^. Studies investigating RNA modifications, particularly m^6^A, in the innate immune response are emerging. Work exploring the role of METTL3 has revealed its involvement in macrophage activation^47^, in the contribution of tumour-associated macrophages to the establishment of the tumour microenvironment^48^ and in the maintenance of macrophage homeostasis during disease progression^49^. However, many aspects remain largely unexplored, particularly the implications of multiple RNA modifications (i.e. m^6^A and 5hmC) on gene expression in macrophage development and function.

In this data descriptor, we focused on profiling two RNA modifications, m^6^A and 5hmC, in the context of macrophage differentiation and polarisation. We present a multi-omics dataset featuring five regulatory layers within the gene expression process (transcriptome, transcriptome-wide mapping of m^6^A and 5hmC, translatome and proteome) of 4 cellular states (monocytes, resting-like macrophages and pro- and anti-inflammatory-like macrophages) modelling macrophage differentiation and polarisation. The experimental approach that we took aimed to generate paired datasets that would allow us to derive hypotheses on the functions of RNA modifications as regulators of gene expression in macrophages. The main strength of this dataset is that the same RNA preparation was used for mRNA-Seq, m^6^A- and 5hmC-IP-Seq facilitating their integration for analysis and interpretation. While m^6^A has been mapped in a variety of tissues and conditions including mouse macrophages^33,47^ to the best of our knowledge, only two transcriptome-wide RNA-5hmC maps have been published to date, one performed in Drosophila cells^29^ and one in mouse embryonic stem cells^30^. Therefore, the dataset we present here will be a useful reference for validation studies using novel technologies such as Nanopore sequencing that will allow the simultaneous detection of different RNA modifications.

This rich dataset, generated in the context of a highly- dynamic process offers a unique resource to study, for example, the impact of environmental cues on RNA modification patterns and to explore potential crosstalk and/or interplay between different modifications. It will facilitate the building of novel hypotheses to determine the roles of m^6^A and 5hmC across the gene expression process. We have previously published a subset of this dataset, namely mRNA-Seq and LC-MS/MS for monocytes, resting-like macrophages and pro-inflammatory-like macrophages, elsewhere^50^.

## Methods

### Experimental design

**Fig. 1:**
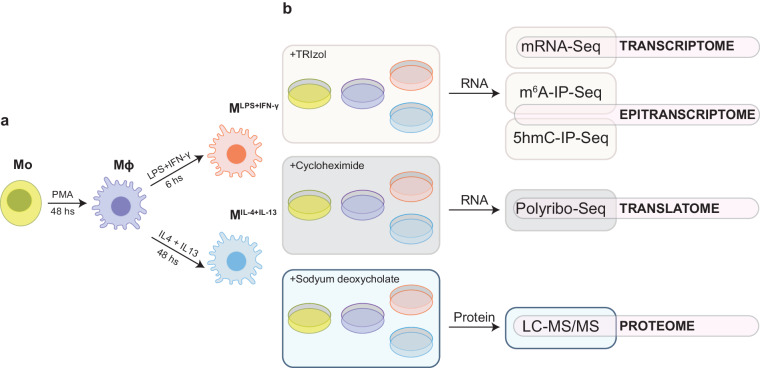
Schematic overview and experimental design. (**a**) Macrophage differentiation and polarisation strategy. Mo: THP-1 monocytes, Mϕ: resting-like macrophages, M^LPS+IFN-γ^: Pro-inflammatory-like macrophages, M^IL-4+IL-13^: anti-inflammatory-like macrophages. (**b**) Multi-omics dataset generation strategy. mRNA-Seq, m^6^A-IP-Seq and 5hmC-IP-Seq were performed in parallel, with the same extracted RNA.

Figure 1 illustrates the experimental design behind the dataset presented here. Panel a presents the strategy used to model THP-1 macrophage differentiation and polarisation *in vitro* as previously described by us and others^50,51^. Panel b summarizes the approach taken to generate the individual omics datasets for THP-1 monocytes (Mo), resting-like (Mϕ), pro-inflammatory-like (M^LPS+IFN-γ^) and anti-inflammatory-like (M^IL-4+IL-13^) macrophages.

#### THP-1 differentiation and polarisation

A large batch of low-passage THP-1 cells (expanded from ATCC, TIB‐202) was cultured and used to generate the entire dataset. THP-1 cells were maintained below 1 million cells per ml in RPMI medium (Thermo Fisher Scientific) supplemented with 10% (v/v) foetal calf serum (Hyclone, GE Healthcare), 1% (v/v) non-essential amino acids (Thermo Fisher Scientific), 1 mM sodium pyruvate (Thermo Fisher Scientific) and 0.1 mg/ml penicillin and streptomycin (Thermo Fisher Scientific) at 37 °C, 5% CO_2_. To generate THP-1-derived macrophages (Mϕ), THP-1 monocytes (Mo) were stimulated with 100 nM phorbol-12-myristate 13-acetate (PMA, Sigma) and 50 μM 2-mercaptoethanol for 48 hours. M^LPS+IFN-γ^ macrophages were generated by stimulating Mϕ with 1 μg/ml Lipopolysaccharide (LPS, Sigma) and 20 ng/ml IFN-γ for 6 hours. M^IL-4+IL-13^ macrophages were generated by stimulating Mϕ with 20 ng/ml IL-4 (R&D Systems) and 20 ng/ml IL-13 (R&D system) for 48 hours.

This experiment was performed three times. From the first experiment, RNA was extracted, and the same preparation was used for mRNA-Seq, m^6^A-IP-Seq and 5hmC-IP-Seq; from the second experiment, RNA was extracted for Polyribo-Seq and from the third experiment protein was extracted for liquid-chromatography tandem mass spectrometry.

#### Acquisition and pre-processing of omics data

##### RNA extraction

Total RNA was extracted using TRIzol Reagent (Invitrogen). A maximum of 10 million cells were lysed per 1 ml of TRIzol reagent and incubated for 10 minutes at room temperature. 200 μl were added to the lysate, shaken vigorously, and incubated for 15 minutes at room temperature prior to centrifugation for 20 minutes at 12,000 g, 4 °C. The aqueous phase was then transferred to a tube containing 500 μl of isopropanol and 1 μl of glycogen (Invitrogen) and precipitated at −30 °C overnight. Next, samples were centrifuged for 20 minutes at 12,000 g, 4 °C. The supernatant was discarded, and the pellet was washed with 1 ml of 75% ethanol. Samples were centrifuged for 5 minutes at 7,000 g, 4 °C, the supernatant discarded, and the pellet air-dried for approximately 5 minutes. The pellets were resuspended in 50 μl of RNAse-free water. To remove possible genomic DNA contamination, RNA was treated with TURBO DNAse (Thermo Fisher Scientific). A maximum of 200 μg of RNA per ml was added to a mixture containing 0.1 volume of 1x TURBO DNase buffer and 1 μl of TURBO DNAse and incubated at 37 °C for 20 minutes. 0.1 volume of DNAse inactivation reagent was added to the sample, incubated and mixed for 5 minutes at room temperature. Next, samples were centrifuged for 2 minutes at 10,000 g, at room temperature to pellet the inactivation reagent. RNA was transferred to a new tube. RNA concentration was determined using nanodrop and/or Qubit. RNA integrity was determined using RNA Nano 6000 Bioanalyzer (Agilent).

##### Transcriptomics

We have previously described a subset of the transcriptomics dataset (Mo, Mϕ and M^LPS+IFN-γ^) and this section of the method has been peer-reviewed^50^.

##### mRNA-Seq

**Fig. 2:**
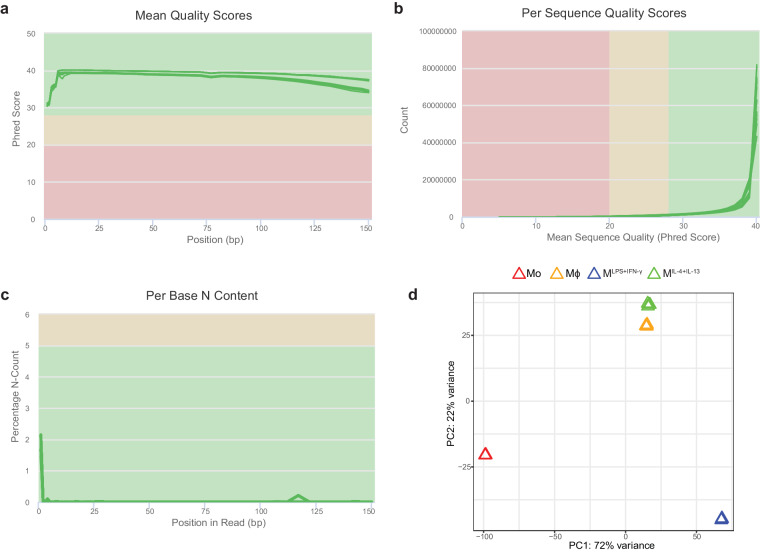
Quality control and clustering analysis of the mRNA-Seq dataset. (**a**) Mean quality scores across each base position in the read expressed as Phred score for all samples. (**b**) Quality score distribution over all reads obtained for all samples. (**c**) Percentage of bases at each position with no base call (N). Green lines represent individual samples. The background colour of each graph indicates whether the region is bad (red), acceptable (yellow) or good (green). (**d**) Principal component analysis of gene expression profiles.

2 μg of total RNA per sample was sent to a commercial sequencing facility (Novogene, China) for paired-end mRNA-Seq. Following directional mRNA library preparation (mRNA enrichment) samples were sequenced using the NovaSeq system (Illumina). Approximately 200 million 150 bp paired-end reads were obtained from each sample (Supplementary Table 1). This experiment was performed in triplicates for each condition. Data quality of the raw sequencing data from fastQC was merged using MultiQC^52^. Good quality was observed across all samples including mean quality scores (Fig. 2a), per sequence quality scores (Fig. 2b) and per base N content (Fig. 2c). Truseq3-PE adapter and poor-quality sequences were trimmed using Trimmomatic^53^ using the default settings. Trimmed reads were then aligned to the human reference genome hg38 (ENSEMBL version 86) using STAR aligner^54^. FeatureCounts^55^ was then used to convert aligned short reads into read counts for each sample. The number of mapped reads was uniform across the dataset (Supplementary Table 1). R and DESeq2^56^ were used to analyse the data. Expressed genes were identified as those with RPKM greater than 1 for at least one group of samples. Differentially expressed genes (DEGs) between two groups were identified using Wald statistics, with fold-change > 1.5 and *p* < 0.05 after Benjamini-Hochberg correction. Lists of DEGs are available in (Supplementary Table 2). Differences in global gene expression patterns between Mo, Mϕ, M^LPS+IFN-γ^ and M^IL-4+IL-13^ and sample variance were evident by principal component analysis (PCA) plotting (Fig. 2d).

#### Epitranscriptomics

##### m^6^A-IP-Seq

m^6^A-IP-Seq was performed as previously described with some modifications^57,58^. 5 μg of DNAse treated total RNA was fragmented by treatment with 100 mM Tris-HCl, 100 mM ZnCl_2_ in nuclease-free H_2_O at 70 °C for 13 minutes in a thermocycler (71 °C lid temperature). The reaction was stopped by the addition of 0.5 M EDTA. Fragmented RNA was precipitated by incubation with RNAse free 3 M sodium acetate pH 5.2 (Sigma), glycogen (Invitrogen) and 100% ethanol at −80 °C overnight. Fragment-size distribution was assessed using RNA 6000 Nano Bioanalyzer kit (Agilent). Fragmentation time was optimised to achieve a fragment size distribution peaking at 100–150 nt with more than 95% of all fragments between 50–500 nt in length for all samples. Approximately 500 ng of sample were stored at −80 °C as input control. Fragmented RNA was subjected to two rounds of m^6^A immunoprecipitation for 2 hours each using an anti-m^6^A antibody (ABE572, Merck) previously conjugated to protein-A magnetic beads (Thermo Fisher Scientific) and of protein-G magnetic beads (Thermo Fisher Scientific). Bead-antibody conjugation was achieved by incubation of 30 μl of each of the beads with 5 μg of anti-m^6^A antibody in IP buffer (150 mM NaCl, 10 mM Tris-HCl, pH 7.5, 0.1% IGEPAL CA-630 in nuclease free H_2_O) at 4 °C for at least 6 hours. After washing the antibody-beads mixture with IP buffer twice, this was added to a mixture containing the fragmented RNA, RNAsin Plus (Promega) and IP buffer and incubated for 2 hours at 4 °C. All incubations involving beads and antibody were performed at 4 °C in a rotating platform. Using a magnetic rack, the low/high salt washing method (two washes in IP buffer, two washes in low-salt IP buffer (50 mM NaCl, 10 mM Tris-HCl, pH 7.5, 0.1% IGEPAL CA-630 in nuclease free H_2_O), and two washes in high-salt IP buffer (500 mM NaCl, 10 mM Tris-HCl, pH 7.5, 0.1% IGEPAL CA-630 in nuclease free H_2_O) for 10 min each at 4 °C) was used. Next, the m^6^A-enriched RNA was eluted from the beads using RLT buffer and the RNeasy mini kit (Qiagen) as per the manufacturer’s instructions. RNA was quantified using RNA 6000 Pico Bioanalyzer kit (Agilent). Finally, library preparation was performed using the SMARTER Stranded Total RNA Seq kit v2-Pico Input Mammalian kit (Takara Bio) following the manufacturer’s instructions. m^6^A-enriched samples were amplified for 16 cycles and input samples were amplified for 12 cycles. Library quality was assessed by running a DNA Bioanalyzer chip (Agilent). This experiment was performed in duplicates for each condition.

**Fig. 3:**
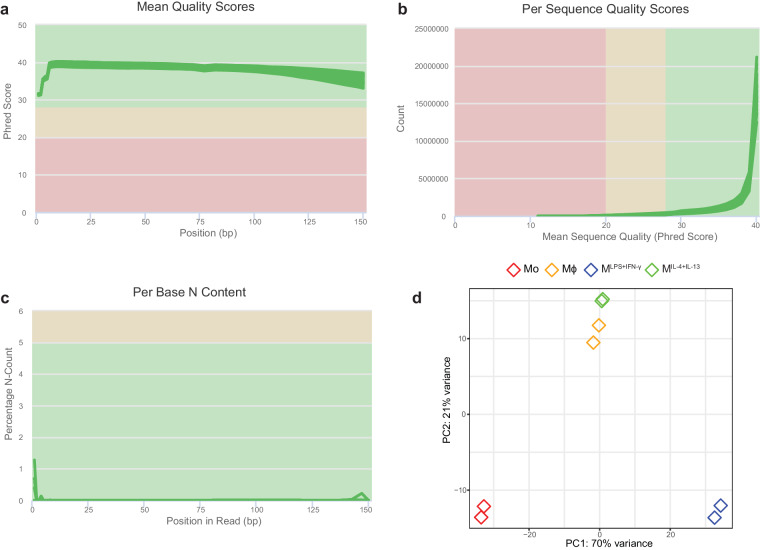
Quality control and clustering analysis of the m^6^A-IP-Seq dataset. (**a**) Mean quality scores across each base position in the read expressed as Phred score for all samples. (**b**) Quality score distribution over all reads obtained for all samples. (**c**) Percentage of bases at each position with no base call (N). Green lines represent individual samples. The background colour of each graph indicates whether the region is bad (red), acceptable (yellow) or good (green). (**d**) Principal component analysis of transcriptome-wide m^6^A profiles.

Samples were sent to a commercial sequencing facility (Novogene, China) for sequencing using the Illumina NovaSeq system. A minimum of 60 million paired-end reads were obtained per sample (Supplementary Table S1). Raw sequencing data quality was assessed using fastQC and good quality was observed in all cases (Fig. 3a–c). Raw reads were trimmed using Trimmomatic^53^ to remove the adapters using the default settings. Cleaned reads were then aligned to the human reference genome hg38 (ENSEMBL version 86) using STAR aligner^54^. Only uniquely mapped reads were selected using samtools^59^ to minimise the rate of false positives. Peaks enriched in immunoprecipitated over corresponding input samples were called using MACS2^60^. The number of m^6^A peaks identified for each sample and replicate are listed in Supplementary Table 3. Peaks identified in both biological replicates were merged using the mergePeaks command in the HOMER software^61^ and overlapping peaks were mapped to the RefSeq gene annotation using intersectBed from BEDTools^62^. For differential methylation analysis purposes, overlapping peaks identified in each condition were merged using the mergePeaks command. Peak counts were normalised through DESeq2 negative binomial distribution model. Differences in transcriptome-wide m^6^A patterns between Mo, Mϕ, M^LPS+IFN-γ^ and M^IL-4+IL-13^ and sample variance were evident by PCA analysis (Fig. 3d). Gene ontology (GO) biological processes (BP) enrichment analysis was performed for the genes with significantly increased/decreased m^6^A peaks using the ‘clusterProfiler’^63^ package. Enriched m^6^A motifs were identified using *de novo* motif search with the HOMER software (version 4.9.1). Motifs with the most significant *P*-values were visualised using WebLogo^64^. The metagene profiles were plotted using the ‘Guitar’^65^ R package.

##### 5hmC-IP-Seq

**Fig. 4:**
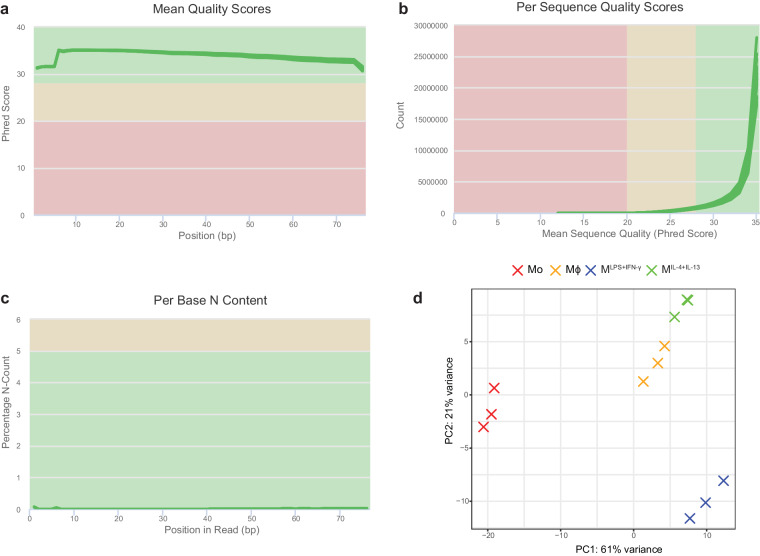
Quality control and clustering analysis of the 5hmC-IP-Seq dataset. (**a**) Mean quality scores across each base position in the read expressed as Phred score for all samples. (**b**) Quality score distribution over all reads obtained for all samples. (**c**) Percentage of bases at each position with no base call (N). Green lines represent individual samples. The background colour of each graph indicates whether the region is bad (red), acceptable (yellow) or good (green). (**d**) Principal component analysis of transcriptome-wide 5hmC profiles.

5hmC-IP-Seq was performed as previously described^29^. 1 mg of DNAse-treated total RNA was fragmentated by incubation in 10 mM Tris-HCl pH7, 100 mM ZnCl_2_ at 94 °C for 40 seconds. The reaction was stopped using 50 mM EDTA. Fragmented RNA was ethanol-precipitated and resuspended in nuclease-free water. Fragment-size distribution was assessed using an RNA 6000 Nano Bioanalyzer kit (Agilent). Prior to immunoprecipitation, fragmented RNA was denatured by incubation at 70 °C for 5 minutes and placed on ice. Fragmented RNA was subjected to 5hmC immunoprecipitation by incubation with 12.5 μl of anti-5hmC antibody (C15220001, Diagenode) in IP buffer (50 mM Tris-HCl, pH 7.4, 750 mM NaCl, 0.5% IGEPAL CA-63, RNAsin 400 U/ml and RVC 2 mM) supplemented with protease inhibitor (complete EDTA free, Roche) at 4 °C overnight. Following the addition of 60 μl of equilibrated Dynabeads Protein G (Invitrogen) samples were incubated for 2.5 hours at 4 °C. After washing the antibody-beads mixture with IP buffer twice, the 5hmC-enriched RNA was eluted by the addition of 1 ml of TriPure Reagent (Roche) as per manufacturer’s instructions. Following reverse transcription of 5hmC-enriched RNA and synthesis of a second strand (NEBNext mRNA second strand synthesis module, NEB), library preparation was performed using the TruSeq ChIP Sample Prep kit (Illumina). 5 to 10 μg of dsDNA were subjected to 5′ and 3′ protruding end repair. To allow ligation of Illumina multiplex adapters, non-templated adenines were added to the 3’-ends of the blunted DNA fragments. The DNA fragments were size selected to remove unligated adapters and to sequence fragments of 200–300 bp of length. The library was amplified through 18 PCR cycles. DNA was quantified using Qubit and DNA integrity was assessed by running a DNA Bioanalyzer chip (Agilent). This experiment was performed in triplicates for each condition. 1.5 pM of DNA library spiked with 1% PhiX viral DNA was clustered and sequenced on a NextSeq 500 (Illumina). A minimum of 40 million paired-end reads were obtained per sample (Supplementary Table 1). As for m^6^A-IP-Seq, raw sequencing data quality was assessed using fastQC and good quality was observed in all cases (Fig. 4a–c). Raw reads were trimmed using Trimmomatic^53^ to remove the adapters using the default settings. Cleaned reads were then aligned to the human reference genome hg38 (ENSEMBL version 86) using STAR aligner^54^. Only uniquely mapped reads were selected using samtools^59^ to minimise the rate of false positives. Peaks enriched in immunoprecipitated over corresponding input samples were called using MACS2^60^. The number of 5hmC peaks identified for each sample and replicate are listed in Supplementary Table 3. Peaks identified in all biological replicates were merged using the mergePeaks command in the HOMER software^61^ and overlapping peaks were mapped to the RefSeq gene annotation using intersectBed from BEDTools^62^. For differential methylation analysis purposes, overlapping peaks identified in each condition were merged using the mergePeaks command. Peak counts were normalised through DESeq2 negative binomial distribution model. Differences in transcriptome-wide 5hmC patterns between Mo, Mϕ, M^LPS+IFN-γ^ and M^IL-4+IL-13^ and sample variance were evident by PCA analysis (Fig. 4d). Gene ontology (GO) biological processes (BP) enrichment analysis was performed for the genes with significantly increased/decreased 5hmC peaks using the ‘clusterProfiler’^63^ package. Enriched 5hmC motifs were identified using *de novo* motif search with the HOMER software (version 4.9.1). Motifs with the most significant *P*-values were visualised using WebLogo^64^. The metagene profiles were plotted using the ‘Guitar’^65^ R package.

#### Translatomics

##### Polyribo-Seq

Polyribosomes were separated by μHPLC Size Exclusion Chromatography (SEC) as previously described^66^ with some modifications.

##### Preparation of cell lysates for SEC

For Mo, 10 million cells per replicate were pelleted and then resuspended in ice-cold PBS containing 50 μg/ml cycloheximide and incubated for 2 minutes at room temperature. Cells were then pelleted, snap-frozen and stored at −80 °C to be processed with the other samples. For Mϕ, M^LPS+IFN-γ^ and M^IL-4+IL-13^, 10 million cells were differentiated and/or polarised as described above. Then, culture media was replaced by ice-cold PBS containing 50 μg/ml cycloheximide and incubated for 2 minutes at room temperature. Cells were then incubated with Accutase (Thermo Fisher Scientific) supplemented with 50 μg/ml cycloheximide at 37 °C, 5% CO_2_ for 30 minutes for detaching. Cells were then harvested and washed with ice-cold PBS containing 50 μg/ml cycloheximide. Cell pellets were snap-frozen and stored at −80 °C to be processed with the other samples.

Cells were lysed on ice in lysis buffer (20 mM HEPES-NaOH (pH 7.4), 130 mM NaCl, 10 mM MgCl_2_, 1% CHAPS, 0.2 mg/ml heparin, 2.5 mM DTT, 50 ug/ml cycloheximide, 20 U SUPERase In RNAse inhibitor (Thermo Fisher Scientific), complete EDTA-free Protease inhibitor (Roche)), incubated on ice for 15 minutes and then centrifuged at 17,000 g for 10 minutes at 4 °C. Supernatants were filtered through 0.45 um Ultrafree-MC HV centrifugal filter units by centrifugation at 12,000 g for 10 minutes at 4 °C. Protein and RNA amounts in the filtrates were quantified by BCA protein assay (Micro BCA Protein Assay Kit, Thermo Fisher Scientific) and Qubit RNA Broad Range assay kit (Thermo Fisher Scientific) respectively. Filtrates were diluted to a protein concentration of 10 mg/ml to inject 1 mg of protein (100 μl per injection).

##### SEC

**Fig. 5:**
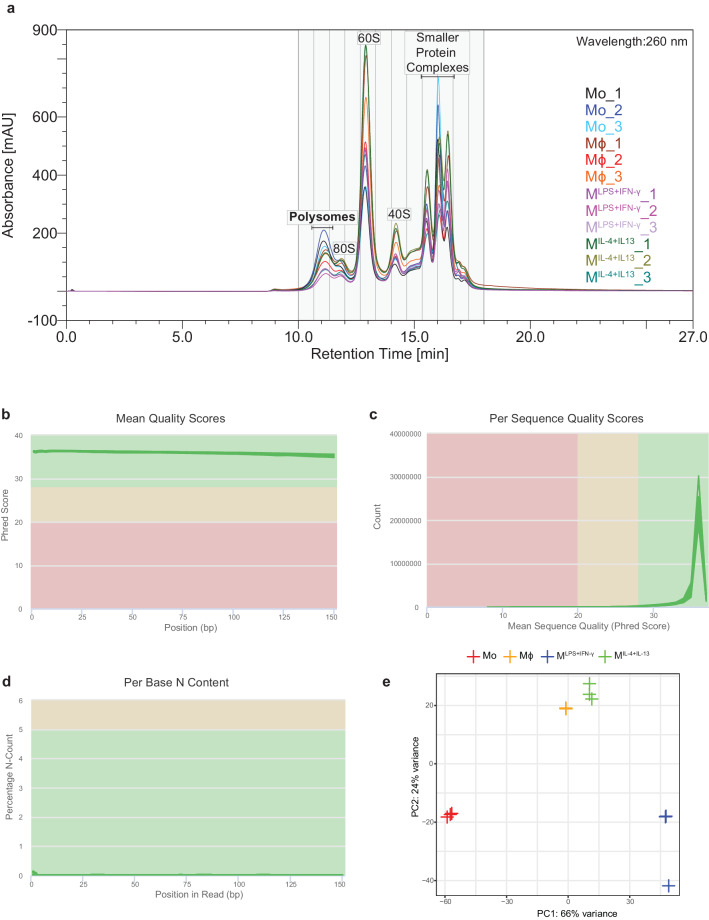
Quality control and clustering analysis of the Polyribo-Seq dataset. (**a**) The ultraviolet chromatograms of THP-1 Mo, Mϕ, M^LPS+IFN-γ^ and M^IL-4+IL-13^ cell lysates. The polysome fraction sequenced is indicated (**b**) Mean quality scores across each base position in the read expressed as Phred score for all samples. (**c**) Quality score distribution over all reads obtained for all samples. (**d**) Percentage of bases at each position with no base call (N). Green lines represent individual samples. The background colour of each graph indicates whether the region is bad (red), acceptable (yellow) or good (green). (**e**) Principal component analysis of translatomic profiles.

For SEC LC analysis a Thermo Dionex BioRs μHPLC system (Thermo Fisher Scientific) and an Agilent SEC-5 7.8 × 300 mm HPLC column with 2000Å pores and 5 mm particles were used. The column was preequilibrated using two column volumes of 0.45 um filtered polysome SEC buffer (20 mM HEPES-NaOH (pH 7.4), 60 mM NaCl, 10 mM MgCl_2_, 0.3% CHAPS, 0.2 mg/ml heparin, 2.5 mM DTT) and 100 μl of a 10 mg/ml solution of BSA (bovine serum albumin) in PBS was injected once to block non-specific interactions. The column conditions were monitored by injecting 10 μl of 10 mg/ml BSA solution and 25 μl of HyperLadder 1KB (Bioline) standards. Chromatograms were monitored at UV absorbances of 215, 260 and 280 nm with 1 Hz of data collection rate by the Diode Array Detector (Fig. 5a). Fractions were collected at a flow rate of 0.8 mL/min from 9 minutes to 14.6 minutes into low protein binding 96-deep-well 1 ml plates (Eppendorf) at 4 °C.

##### Polyribo-Seq

RNA was extracted from the polysomal fraction using TRIzol LS reagent (Thermo Fisher Scientific) following the manufacturer’s instructions. 2 μg of total RNA per sample was sent to a commercial sequencing facility (Novogene, China) for ribosomal RNA depletion, stranded-specific library preparation using the Illumina Stranded Total RNA Prep with Ribo-Zero Plus Kit (Illumina) as per manufacturer’s instructions. Sequencing was performed using the Illumina NovaSeq system. Approximately 60 million paired-end 150 bp reads were obtained from each sample (Supplementary Table 1). Raw sequencing data quality was assessed using fastQC and good quality was observed in all samples (Fig. 5b–d). Truseq3-PE adapter and poor-quality sequences were trimmed using Trimmomatic applying the default settings^53^. Trimmed reads were then aligned to the hg38 (ENSEMBL version 86) human reference genome using STAR aligner^54^. FeatureCounts^55^ was then used to convert aligned short reads into read counts for each sample. The number of mapped reads was uniform across the dataset (Supplementary Table 1). R and DESeq2^56^ were used to analyse the data. Polysome-bound or actively translated genes were identified as those with RPKM greater than 1 for at least one group of samples. Differentially translated genes (DTGs) between two groups were identified using Wald statistics, with fold-change > 1.5 and *p* < 0.05 after Benjamini-Hochberg correction, lists of DTGs are available in Supplementary Table 4. Differences in global translational patterns between Mo, Mϕ, M^LPS+IFN-γ^ and M^IL-4+IL-13^ and sample variance were evident by PCA analysis (Fig. 5e).

##### Proteomics

We have previously described a subset of the proteomics dataset in this method section (Mo, Mϕ and M^LPS+IFN-γ^) that has been peer-reviewed^50^.

##### Preparation of cell lysates for LC-MS/MS

This experiment was performed in triplicates as described previously^50,67^. For Mo, 10 million cells per replicate were pelleted, washed with PBS and lysed by resuspending in SDC denaturing lysis buffer (4% sodium deoxycholate, 100 mM Tris-HCl (pH 8.0)) and immediately heated at 95 °C for 10 minutes. For Mϕ, M^LPS+IFN-γ^ and M^IL-4+IL-13^, 10 million cells were differentiated and/or polarised as described above. Then, culture media was removed, and cells were washed with PBS before incubation for 30 minutes in Accutase (Thermo Fisher Scientific) at 37 °C, 5% CO_2_ for detaching. Cells were then harvested, pelleted and washed with PBS. Cells were lysed by resuspension in SDC denaturing lysis buffer and immediately heated at 95 °C for 10 minutes. Lysates were then sonicated for 10 minutes of total sonication time at 30 seconds on/30 seconds off cycles, 20% amplitude at room temperature using a QSonicaQ800R2 sonicator. After clarifying the lysates by centrifugation at 180,000 g for 10 minutes at 18 °C, protein concentration was measured using the BCA protein assay (Micro BCA Protein Assay Kit, Thermo Fisher Scientific). To denature, reduce and alkylate proteins, 20 μg of protein lysate was taken to a final volume of 25 μl in SDC buffer (4% sodium deoxycholate, 10 mM TCEP, 40 mM chloroacetamide and 100 mM Tris–HCl (pH 8.5) and heated at 95 °C for 10 minutes. After allowing to cool down to room temperature, samples were diluted 4-fold with 75 μl of water and trypsin was added (from 1 mg/ml stock in 50 mM acetic acid) at a 1:20 protein:protease (μg/μg) ratio and digested at 37 °C for 16 hours. An equal volume of 99% ethylacetate/1% TFA was added to the digested peptides and vortexed. To generate SDB-RPS StageTips, double-stacked SDB-RPS discs (Sigma) were punched with an 18-gauge needle and mounted in 200 μl tips (Eppendorf). For clean-up utilising the Spin96, each tip was wetted with 100 μl of 100% acetonitrile and centrifuged at 1000 g for 1 minute. Following wetting, each StageTip was equilibrated with 100 μl of 0.1% TFA in water and 30% methanol/1% TFA with centrifugation for each at 1000 g for 3 minutes. Each StageTip was loaded with ∼20 μg peptide in 100 μl of the lower aqueous phase. The peptides were washed twice with 100 μl of 99% ethylacetate/1% TFA, followed by one wash with 100 μl of 0.2% TFA in water. To elute, 100 μl of 5% ammonium hydroxide/80% acetonitrile was added to each tip and centrifuged as above for 5 minutes into an unskirted PCR plate. Samples were dried in the PCR plate using a GeneVac EZ-2 using the ammonia setting at 35 °C for 1 hour. Dried peptides were resuspended in 60 μl of 5% formic acid and stored at 4 °C until analysed by LC–MS/MS.

##### LC-MS/MS and analysis of spectra

**Fig. 6:**
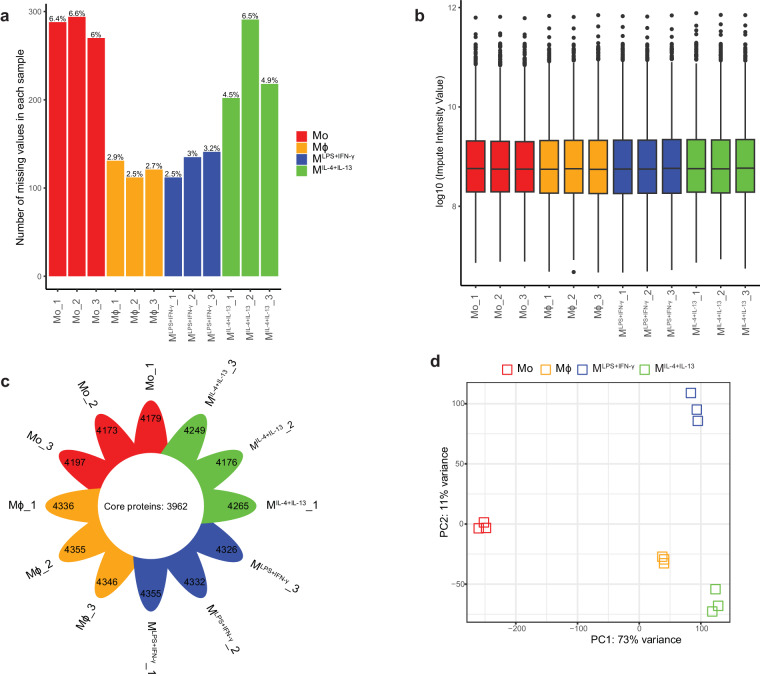
Quality control and clustering analysis of the LC-MS/MS dataset. (**a**) Percent and count of the missing values in each sample. (**b**) Distribution of the quantitative data after ‘missing value’ imputation within samples. (**c**) Count of common proteins within samples. (**d**) Principal component analysis of proteomic profiles.

Peptides in 5% (vol/vol) formic acid (injection volume 3 μl) were directly injected onto a 50 cm × 75 μm C18 (Dr Maisch, Ammerbuch, Germany, 1.9 μm) fused silica analytical column with a ∼10 μm pulled tip coupled online to a nanospray ESI source, using a Thermo Fisher RSLCnano μHPLC. Peptides were resolved over gradient from 5% acetonitrile to 40% acetonitrile over 140 minutes with a flow rate of 300 nl min^−1^ and ionised by electrospray ionisation at 2.3 kV. Tandem mass spectrometry analysis was performed on a Fusion Lumos mass spectrometer (Thermo Fisher Scientific) using HCD fragmentation. The data-dependent acquisition method used acquired MS/MS spectra of the top 20 most abundant ions at any one point during the gradient. The MaxQuant^68^ (version 1.6.3.4) quantitative proteomics software was used to analyse raw data. Peptide and protein level identification were both set to a false discovery rate (FDR) of 1% using a target-decoy-based strategy. Peptide identification was performed using the integrated MaxQuant Andromeda^69^ search engine. The database supplied to the search engine for peptide identification contained the human UniProt database downloaded on 14th August 2018. Mass tolerance was set to 4.5 ppm for precursor ions and MS/MS mass tolerance was 20 ppm. Enzyme specificity was set to trypsin, with a maximum of 2 missed cleavages permitted. Deamidation of Asn and Gln, oxidation of Met, pyro-Glu and protein N-terminal acetylation were set as variable modifications. Carbamidomethyl on Cys was searched as a fixed modification. The MaxLFQ algorithm was used for label-free quantitation, integrated into the MaxQuant environment^68,70^. The MaxQuant output was processed and analysed using R software. The missing values of each sample were evaluated by is.na() R function and the high proportion of missing values were filtered. A total of 4467 proteins were retained with an average missing rate of 4.3% (Fig. 6a). Imputation of missing values was performed using the mean value of two available replicates, the distribution of processed data is shown in Fig. 6b. The overlapping proteins within the four experimental groups are represented in Fig. 6c,most of the identified proteins were common to the 12 samples. Differentially expressed proteins (DEPs) were identified using edgeR^71^, lists of DEPs are available in Supplemental Table 5. Differences in proteomic patterns between Mo, Mϕ, M^LPS+IFN-γ^ and M^IL-4+IL-13^ and sample variance were evident by PCA analysis (Fig. 6d).

## Data Records

The raw multi-omics data has been deposited in GEO (mRNA-, polyribo-, m6A-IP- and 5hmC-IP-Seq data)^72–75^ and PRIDE (LC-MS/MS data)^76^ public repositories as detailed in Table 1.

**Table 1:** Public repositories hosting the macrophages omics data.

Dataset	Samples	Repository	Accession		Reference
			*Superseries*	*Subseries*	
mRNA-Seq	Mo	GEO	GSE130011		^72^
	Mϕ				
	M^LPS+IFN-γ^				
	M^IL-4+IL-13^				
Polyribo-Seq	Mo	GEO	GSE213207	GSE213204	^73^
	Mϕ				
	M^LPS+IFN-γ^				
	M^IL-4+IL-13^				
m^6^A-IP-Seq (MeRIP-Seq)	Mo	GEO	GSE213207	GSE213206	^74^
	Mϕ				
	M^LPS+IFN-γ^				
	M^IL-4+IL-13^				
5hmC-IP-Seq (hMeRIP-Seq)	Mo	GEO	GSE213207	GSE213203	^75^
	Mϕ				
	M^LPS+IFN-γ^				
	M^IL-4+IL-13^				
Proteomics	Mo	PRIDE	PXD017391		^76^
	Mϕ				
	M^LPS+IFN-γ^				
	M^IL-4+IL-13^				

THP-1 monocytes were labelled Mo, resting-like macrophages were labelled Mϕ, THP-1-pro-inflammatory-like macrophages were labelled M^LPS+IFN-γ^ and THP-1-anti-inflammatory-like macrophages were labelled M^IL-4+IL-13^. Except for M^IL-4+IL-13^, other mRNA-Seq datasets (Mo, Mϕ and M^LPS+IFN-γ^) have been previously published^50^. The poly ribo-Seq dataset is named ‘Translatomes of monocytes and macrophages’ in GEO and has not been previously published. The m^6^A-IP-Seq and 5hmC-IP-Seq datasets named ‘m6A modification of monocytes and macrophages’ and ‘5hmC modification of monocytes and macrophages’ respectively in GEO have not been previously published. For m^6^A-IP-Seq, ‘IP_’ refers to m6A immunoprecipitated samples, while ‘Input_’ refers to non-immunoprecipitated samples. For 5hmC-IP-seq ‘5hmC_’ refers to 5hmC immunoprecipitated samples, while ‘INP_’ refers to non-immunoprecipitated samples. A subset of the proteomics dataset (Mo, Mϕ and M^LPS+IFN-γ^) has been previously published^50^. In all cases, biological replicates were given the designation 1, 2 or 3.

## Technical Validation

### Validation of dataset replicability

**Fig. 7:**
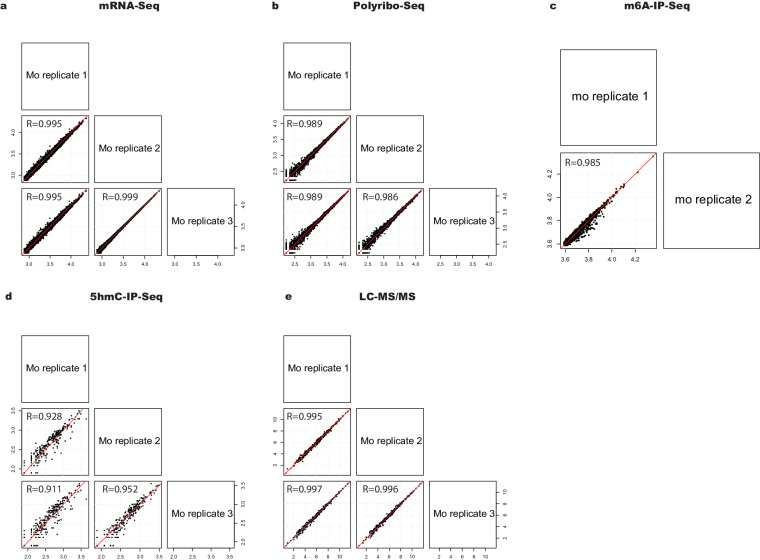
Replicability of processed data. (**a**) mRNA-Seq, (**b**) Polyribo-Seq, (**c**) m^6^A-IP-Seq, (**d**) 5hmC-IP-Seq and (**e**) LC-MS/MS. The red dashed line indicates perfect correlation between samples. Mo samples are presented as an example and Log2 transformed data are shown. Pearson correlation coefficient (R) values are shown.

To assess replicability, pairwise scatterplots comparing replicates were generated for mRNA-Seq, Polyribo-Seq, m6A-IP-Seq, 5hmC-IP-Seq and LC-MS/MS data (Fig. 7a–e). Only Mo datasets are shown as macrophages (Mϕ, M^LPS+IFN-γ^ and M^IL-4+IL-13^) datasets returned similar results. We observed a high correlation between replicates and no experimental outliers were identified.

### Validation of biological consistency

**Fig. 8:**
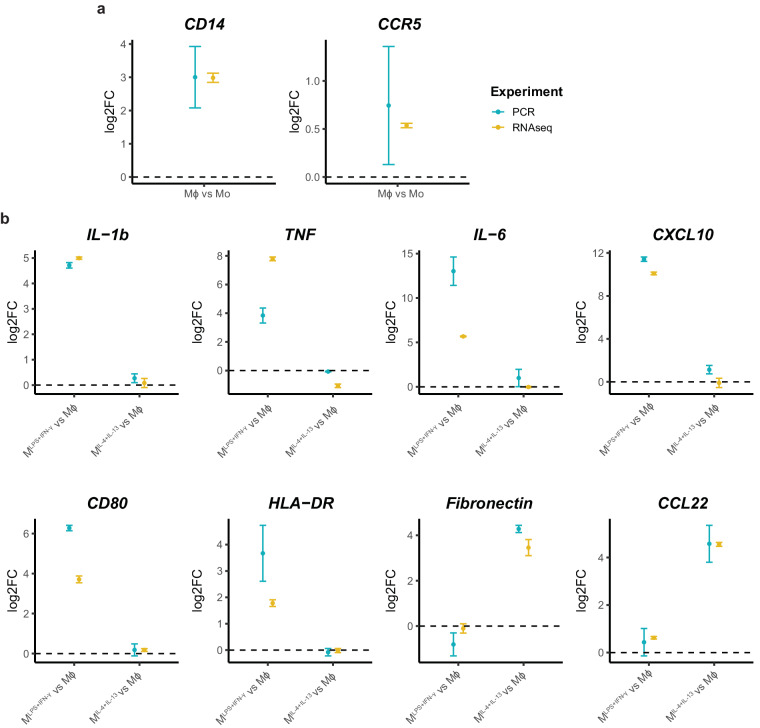
Comparison of differential gene expression of macrophage differentiation and polarisation markers detected by q-RT-PCR and mRNA-Seq. Log2 fold gene expression changes detected by qRT-PCR and mRNA-Seq for (**a**) macrophage differentiation and (**b**) polarisation markers.

**Fig. 9:**
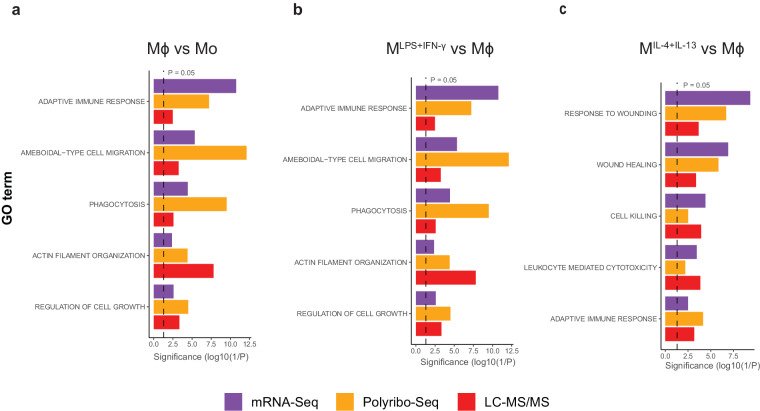
Enriched gene ontology terms of differentially expressed genes. Enriched GO terms between Mo and Mϕ (**a**), Mϕ and M^LPS+IFN-γ^ (**b**) and Mϕ and M^IL-4+IL-13^ (**c**). The black dashed line indicates significance denoted by P-value < 0.05.

To assess biological consistency, we confirmed differential expression of well-established Mϕ macrophage differentiation markers (*CD14* and *CCR5*)^50,77–79^ (Fig. 8a), M^LPS+IFN-γ^ (*IL-1b*, *TNF*, *IL-6*, *CXCL10*, *CD80* and *HLA-DR*) and M^IL-4+IL-13^ (Fibronectin and *CCL22*)^51,80–82^ polarisation markers (Fig. 8b) by qRT-PCR (primers sequences are detailed in Supplementary Table 6) and mRNA-Seq. In all cases, we observed the expected gene expression patterns and both methods were consistent. Furthermore, gene ontology analysis of mRNA-Seq, Polyribo-Seq and LC-MS/MS (Fig. 9a–c) data returned significant enrichment of gene signatures that are characteristic and functionally relevant to the macrophage differentiation and polarisation states. This indicates that the dataset reflects the transcriptomic, translational and proteomic profiles characteristic of differentiated and polarised macrophages.

**Fig. 10:**
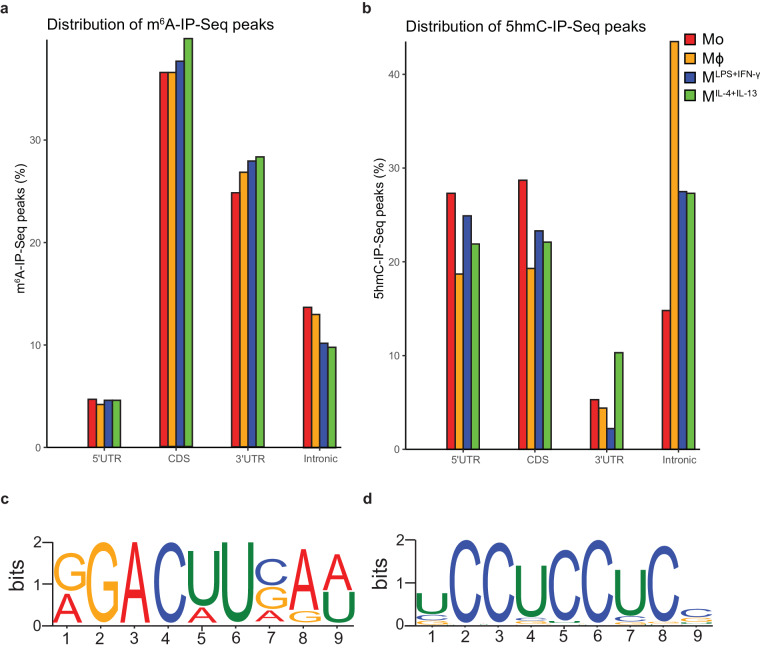
RNA modifications distribution plots and sequence motifs. Distribution of m^6^A-IP-Seq (**a**) and 5hmC-IP-Seq (**b**) peaks. Sequence motif of m^6^A-IP-Seq (**c**) and 5hmC-IP-Seq (**d**) peaks.

To validate the m^6^A-IP-Seq and 5hmC-IP-Seq datasets, we evaluated the distribution of immunoprecipitated peaks and the enrichment for sequence motifs. In line with the literature, we observed an enrichment of m^6^A peaks in the coding sequence (CDS) and towards the 3’UTR which is expected as m^6^A is highly abundant near the stop codon, and as expected, sequence logo analysis returned the m^6^A consensus motif DRACH (D = A, G or U, R = A or G, H = A, C or U)^3,4^ (Fig. 10a,c). Similarly, for 5hmC we obtained results that resembled the distribution of RNA 5hmC peaks and the UC-rich sequence motif previously described by Lan *et al*.^30^ (Fig. 10b,d).

Overall, the technical validation of this dataset shows biological consistency of our mRNA-Seq, Polyribo-Seq and LC-MS/MS datasets with the expected and well-established profiles of modelled differentiated and polarised macrophages. Finally, our m^6^A- and 5hmC-IP_Seq datasets recapitulated the distribution and sequence motifs previously reported in the literature and we therefore conclude that these datasets provide a useful resource to further investigate these RNA modifications in macrophages.

### Supplementary information

Supplementary Tables

## Data Availability

Pre-processing scripts for each of the omics datasets are available at the Github repository (https://github.com/EpiRNAsLab/Multiomics).
